# Bioavailability of Micronutrients From Nutrient-Dense Whole Foods: Zooming in on Dairy, Vegetables, and Fruits

**DOI:** 10.3389/fnut.2020.00101

**Published:** 2020-07-24

**Authors:** Alida Melse-Boonstra

**Affiliations:** Division of Human Nutrition and Health, Wageningen University and Research, Wageningen, Netherlands

**Keywords:** bioavailability, vitamins, minerals, dairy, fruits, vegetables

## Abstract

In order to fully exploit the nutrient density concept, thorough understanding of the biological activity of single nutrients in their interaction with other nutrients and food components from whole foods is important. This review provides a narrative overview of recent insights into nutrient bioavailability from complex foods in humans, highlighting synergistic and antagonistic processes among food components for two different food groups, i.e., dairy, and vegetables and fruits. For dairy, bioavailability of vitamins A, B2, B12 and K, calcium, phosphorous, magnesium, zinc and iodine are discussed, whereas bioavailability of pro-vitamin A, folate, vitamin C and K, potassium, calcium, magnesium and iron are discussed for vegetables and fruits. Although the bioavailability of some nutrients is fairly well-understood, for other nutrients the scientific understanding of uptake, absorption, and bioavailability in humans is still at a nascent stage. Understanding the absorption and bioavailability of nutrients from whole foods in interaction with food components that influence these processes will help to come to individual diet scores that better reflect absorbable nutrient intake in epidemiologic studies that relate dietary intake to health outcomes. Moreover, such knowledge may help in the design of foods, meals, and diets that aid in the supply of bioavailable nutrients to specific target groups.

## Introduction

Historically, the nutritional sciences are built on the study of single nutrients or food components in relation to health outcomes. Although this has been a useful concept when it comes to specific deficiency diseases, the picture became blurry when studying the role of nutrition in complex diseases. The notion that the sum of the parts does not necessarily explain the result of the whole has prompted a shift in focus from single nutrients to whole foods, meals, and dietary patterns. Studying the biological activity of single nutrients in their interaction with other nutrients and food components from whole foods, especially during their stay in the gastro-intestinal tract, helps to better understand the underlying positive and adverse health effects of whole foods, meals, and dietary patterns.

Bioavailability of nutrients from whole foods is a research area that has received ample attention in the past decades, although studies in humans are still limited. Over time, some consensus has been reached on the definition of bioavailability, which is the fraction of an ingested nutrient that becomes available for use and storage in the body (1). In this definition, bioavailability goes beyond mere absorption from the gut and also includes the use and storage (retention) in body tissue. The study of absorption and bioavailability of nutrients from foods in humans requires sophisticated methods that take into account endogenous nutrient losses through the enterohepatic circulation as well as incorporation of nutrients into storage tissue. Use of isotopes, both radio-isotopes and stable isotopes, have greatly improved accuracy and precision of *in vivo* nutrient bioavailability studies, either as a single nutrient or as part of a food, meal, or dietary pattern (2). Although *in vitro* methods are much cheaper and faster than *in vivo* methods, allowing for large numbers and experimental conditions, translation of findings to full body human conditions is still cumbersome (3, 4). Therefore, this review uses only bioavailability data from *in vivo* studies in humans, although the underlying mechanisms of interactions between food components are mostly based on *in vitro* or animal studies.

The main objective of this review is to provide an overview of insights into nutrient bioavailability from complex foods in humans, thereby highlighting the current state of knowledge on synergistic and antagonistic processes among food components. Two different food groups are put in the spotlight for this purpose, i.e., dairy, and fruits and vegetables. Both of these food groups contain a myriad of nutrients, for some of which the bioavailability is now well-understood, whereas others still require further study. Both food groups also contain many bioactive components and have a complex matrix, which affect the kinetics of nutrient release, absorption, and bioavailability. Understanding of these processes will help to better predict the true nutrient value of foods and to incorporate this information into diet scores in the future.

## Milk and Dairy Foods

Dairy refers to food products that have milk—mostly cow's milk—as their main ingredient such as buttermilk, yogurt, cheese, and all closely related products. Dairy is characterized by a relatively high amount of protein and fat, and can therefore make an important contribution to calorie intake unless low-fat alternatives are consumed. Intake of dairy varies greatly between and within world regions, with an estimated average intake of milk (excluding other dairy products) of ~200–240 g per day in Western Europe and North America, ~130–300 g per day in Latin America, ~100–200 g per day in Africa, and 20–150 g in Asia^1^. Despite controversy around the healthfulness of dairy with respect to non-communicable disease risk, scientific evidence consistently points toward either beneficial or neutral effects (5–10). In addition, positive effects on bone mineral density have been found, as well as reduced fracture risk in some populations (9, 11). Such beneficial effects have been attributed to calcium as well as to various other nutrients and bioactive components present in milk (11).

In industrialized countries, dairy stands out as source of calcium, but it also contributes for 20–40% to the intake of vitamins A, B2, B12, and K as well as of phosphorous, magnesium, zinc, and iodine (12– 23)^2^. In the next sections, the absorption and bioavailability of these nutrients from milk and dairy foods will be described, with special attention for calcium.

## Dairy as a Source of Calcium

Dairy is by far the most important source of calcium in the human diet and it has therefore been studied most extensively among the nutrients derived from dairy. Bovine milk contains an average of 120 mg calcium per 100 mL. In Europe and North America, ~75% of dietary calcium is derived from milk and dairy products, with an additional 15% from vegetables and fruits, 5% from mineral water and the rest from other foods (24, 25). Approximately 40% of calcium from dairy sources is absorbed under normal circumstances, with higher absorption in children and lower absorption in elderly (26, 27). In the body, 99% of calcium is present in the skeleton. The efficiency of calcium storage in bone tissue is mainly determined by physiological factors (e.g., related to growth, pregnancy, and lactation), and is regulated by several hormones, such as PTH, calcitonin, calcitriol, and estrogens. Excessive absorbed calcium is excreted in urine, feces, and sweat. Adults are generally in negative calcium balance after their peak bone mass (~35 y) and loose ~10 mg of calcium each day, although in post-menopausal women the daily loss may be 40 mg per day or more (28). Bioavailability of calcium is determined by absorption in the small intestine on the one hand and by incorporation into bone tissue on the other hand. Both of these processes can be influenced by dietary factors. The bioavailability of calcium may therefore be defined as the fraction of dietary calcium that is absorbed by the intestine and is used for bone mineralization.

Intestinal absorption of calcium mostly happens by passive diffusion, whereas active transport at low and moderate calcium intake is under regulation of vitamin D (24). Fortification of milk with vitamin D2 was shown to enhance calcium absorption (29). A number of milk and dairy components have been found to aid in the passive absorption of calcium (and also of other divalent cations), such as phosphopeptides, casein and whey proteins, lactose and phosphorous (Table 1). Phosphopeptides, which are products of the enzymatic hydrolysis of casein, sequester calcium thereby protecting it from precipitation by anions like phosphates in the intestine (30–32). The same is true for alpha lactalbumin and beta lactoglobulin, both whey proteins (24), and for amino acids such as L-lysine and L-arginine (30). The calcium bound to these amino acids, peptides, and proteins is readily released during digestion by bringing it slowly into solution, which is an important prerequisite for passive diffusion.

**Table 1:** Synergistic and antagonistic effects of dairy food components on calcium bioavailability.

**Dairy food component**	**Synergism/antagonism**	**References**
Vitamin D	↑ active absorption of calcium at low and moderate intake	(24)
Casein and phosphopeptides	↑ passive diffusion by binding and slow release of calcium into solution in the chymus	(30–32)
Whey proteins alpha lactalbumin, beta lactoglobulin	↑ passive diffusion by binding and slow release of calcium into solution in the chymus	(24)
Amino acids (L-lysine, L-arginine)	↑ passive diffusion by binding and slow release of calcium into solution in the chymus	(30)
Lactose	↑ passive diffusion by widening the paracellular spaces in the enteric cell lining	(33)
Phosphorous	↓ absorption by binding into undigestible complexes ↑ re-absorption of calcium in the distal part of the nephron ↑ uptake of absorbed calcium into bone	(24)
Sulfur-containing proteins	↓ calcium balance by inducing hypercalciuria	(34–40)
Fat	↔ formation of insoluble soaps with calcium, but these are dissociated at the low pH of the stomach	(41)

*↑ Indicates an increase; ↓ indicates a decrease; ↔ indicates no effect*.

Lactose also seems to enhance calcium absorption, although the mechanism has long been unclear (42). The most likely explanation is that, like other sugars, lactose widens the paracellular spaces in the enteric cell lining, thereby enhancing passive diffusion (33). However, studies have shown that this mostly happens at relatively high doses of lactose (43, 44), whereas in the amounts present in milk and dairy it is less likely to contribute much to absorption (45, 46). When lactose is hydrolyzed, such as in yogurt, or absent, such as in cheese, calcium absorption does not seem to be affected (47). Nevertheless, lactose seems to be important for calcium absorption in case of high calcium intake in combination with poor solubility, such as seen in babies and the elderly (48, 49). It may be that, similar to galactic-oligosaccharides, lactose functions as a prebiotic and stimulates calcium absorption in the cecum and colon by enhancing the growth of bifidobacteria and thereby maintaining low pH (50, 51). This hypothesis is supported by evidence from lactose-deficient patients, who do not seem to have compromised calcium absorption (52).

Apart from factors that enhance calcium absorption, several dairy components can inhibit the uptake of calcium (Table 1). Protein, especially sulfur-containing protein, has been shown to lead to a negative calcium balance through increased urinary calcium excretion (24). Nevertheless, the conclusion of a working group that more recently reviewed the current evidence linking dietary protein intake with bone health was that, although a high protein diet—either of animal or vegetable origin—is associated with increased urinary calcium excretion, this is more likely due to higher intestinal calcium absorption than to bone resorption (53, 54). Milk fats can form insoluble soaps with calcium; however, these are dissociated at the low pH of the stomach and therefore do not affect calcium bioavailability negatively (41). This explains that calcium from cheese is readily available for absorption despite the high content of saturated long chain fatty acids (24, 55).

Phosphorous plays a dual role in calcium absorption, by, on the one hand, binding calcium and inhibiting its absorption in the small intestine resulting in increased fecal excretion of calcium, and, on the other hand, after being absorbed, by increasing the reabsorption of calcium in the distal part of the nephron or by enhancing the uptake of absorbed calcium into bone (24, 54). The inhibitory properties of phosphorous on intestinal calcium absorption may partly be countered by phosphorylation of lactose, thereby keeping calcium in solution (24). However, this hypothesis has not yet been confirmed (41, 55). Also, the high phosphorous content of milk may counter the hypercalciuria induced by protein (54, 56). The recommended dietary intake ratio for calcium (mg) to phosphorous (mg) ranges from 1:1 to 1.5:1, with ratios <0.5 being associated with decreases in bone mineral density (57). Moreover, excessive intake of phosphorous has been shown to induce the secretion of fibroblast growth factor 23 (FGF-23) from bone, thereby decreasing the formation of 1,25-dihydroxyvitamin D3 and decreasing intestinal calcium absorption (57, 58). Cow's milk provides calcium and phosphorus in a reasonable balanced ratio of ~1.2:1.

Overall, although intestinal absorption of calcium from milk and dairy is very similar compared to other sources such as calcium salts, vegetables, or mineral water, its net effect on calcium retention is generally higher (59) with little difference between the various dairy products (milk, acidified milk, yogurt, skim milk, cream cheese, hard cheeses) (24). Diets including dairy products can therefore be considered as the most optimal calcium-dense option to prevent adverse health effects related to a negative calcium balance.

## Dairy as a Source of Other Nutrients

### Vitamins

#### Vitamin A

The content of vitamin A in dairy ranges between 15 and 50 μg in 100 mL of milk to over 300 μg per 100 g of full-fat cheese (Table 2). Vitamin A occurs in dairy products predominantly as retinyl palmitate (60), but small amounts of ß-carotene can also occur. Little is known about the bioavailability of vitamin A from milk and other dairy products, but one study reports that ~15% of vitamin A from milk is absorbed and this appeared not to be different for fortified milk (60). Moreover, it did not appear to be different for full fat or skim milk, despite the fact that absorption of fat-soluble vitamins is generally regarded to depend on the fat content of a meal for solubilization and stimulation of biliary secretion and for the formation of micelles (60).

**Table 2:** Bioavailability of vitamins and minerals from milk and dairy foods in humans.

**Nutrient**	**PRI/AI**	**Content (per 100 g)**		**Bioavailability (%)**	**Enhancing factors**	**Inhibiting factors**
Vitamin A	650 μg/d	Milk Yogurt Soft cheese Cheese	15 μg 31 μg 200–300 μg >300 μg	15%	Dietary fat	
Vitamin B2	1.6 mg/d	Milk Yogurt Soft cheese Cheese	0.18 mg 0.16 mg 0.30–0.60 mg 0.28 mg	67%	Non-covalent binding to protein	Covalent binding
Vitamin B12	4 μg/d	Milk Yogurt Soft cheese Cheese	0.45 μg 0.25 μg 1.1 μg 2.0 μg	65%	Binding to transcobalamin or casein	Binding to haptocorrin
Vitamin K-2	70 μg/d	Milk Yogurt Soft cheese Cheese	0.7–1.4 μg 0.1–3.3 μg 2.0–2.5 μg 68 μg	Unknown	Dietary fat Fermentation products Long-chain menaquinones (MK_7−9_)	Medium-chain menaquinones (MK_4_)
Calcium	950 mg/d	Milk Yogurt Soft cheese Cheese	120 mg 125–150 mg 400–600 mg 800 mg	40%	Binding to casein and whey peptides Lactose, amino acids Vitamin D (fortification)	Phosphorous Sulfur-containing proteins
Phosphorous	550 mg/d	Milk Yogurt Soft cheese Cheese	100 mg 100–120 mg 300–500 mg >500 mg	Unknown	Binding to casein and whey peptides Binding to phospholipids	Complexing with unbound calcium
Magnesium	300 mg/d	Milk Yogurt Soft cheese Cheese	10 mg 13 mg 15–25 mg >30 mg	24–75%	Binding to casein and whey peptides Lactose	High dosing
Zinc	7.5 mg/d	Milk Yogurt Soft cheese Cheese	0.4 mg 0.4–0.6 mg 2–3 mg 3.4 mg	25–30%	Mild acidic conditions Whey and casein peptides Low molecular ligands (amino acids, organic acids)	
Iodine	150 μg/d	Milk Yogurt Soft cheese Cheese	15 μg 15 μg 20–40 μg 21 μg	90%	Inorganic, unbound	

*PRI, Population Reference Intake; AI, Adequate Intake for adult females. Source: EFSA: https://www.efsa.europa.eu/sites/default/files/assets/DRV_Summary_tables_jan_17.pdf*.

#### Vitamin B2

With 0.18 mg of riboflavin per 100 mL of milk and 0.28 mg per 100 g of cheese, dairy forms an important source of this water soluble vitamin (Table 2). In dairy, riboflavin is mostly bound non-covalently to protein, predominantly as flavin adenine dinucleotide (FAD) and to a lesser extent as flavin mononucleotide (FMN). Milk also contains free riboflavin bound to specific binding proteins (21). Hydrolysis of FAD and FMN to riboflavin by phosphatases in the small intestine is a prerequisite for its carrier-mediated absorption (21). Riboflavin has been reported to be readily bioavailable from milk at ~67% (61).

#### Vitamin B12

Milk contains ~0.40–0.45 μg of vitamin B12 per 100 mL, whereas cheese can contain up to 2 μg per 100 g (Table 2). The major derivatives of vitamin B12 in bovine milk are hydroxycobalamin, adenosylcobalamin, and methylcobalamin, and it is mostly bound to the proteins haptocorrin, transcobalamin and casein depending on the cow breed (62, 63). Vitamin B12 bound to transcobalamin appeared to be better released *in vitro*, whereas this was cumbersome when bound to haptocorrin (mainly present in buffalo milk) and this may have implications for *in vivo* bioavailability (63). A study in healthy adults > 60 y old, however, revealed that ~65% of vitamin B12 from milk was absorbed (64), whereas, in comparison, absorption of vitamin B12 from animal foods is generally 50% or lower and even <5% for synthetic supplements (62, 65). Nevertheless, a study comparing cyano-B12 from a supplement with hydroxo-B12 from whey powder improved vitamin B12 status similarly (66).

#### Vitamin K-2

Menaquinones are primarily synthesized by bacteria, and therefore fermented dairy products such as yogurt and cheese are good sources of this vitamin (67). Milk contains ~0.7–1.4 μg of menaquinones per 100 mL, whereas full-fat hard cheese can contain up to 68 μg per 100 g (Table 2). Intake of long-chain menaquinones (MK_n_) in particular has been associated with decreased risk of cardiovascular disease (68, 69), in contrast to phylloquinone (vitamin K-1) derived from plant-based foods. The menaquinone content of dairy products was assessed to be highest in fermented cheeses and to be positively related to fat content (70, 71). Vitamin K is a fat soluble vitamin, and as such is absorbed through the lipid pathway. The bioavailability of menaquinones from dairy sources has not been studied in humans to date, with the exception of a study showing that MK_7_-fortified yogurt resulted in slightly higher plasma concentrations as compared to MK_7_ from a soft-gel capsule (72). The length of the isoprene chain strongly determines absorption and metabolism of menaquinones in the sense that MK_7−9_ are better absorbed than MK_4_ and have a longer half-life than vitamin K-1 (67, 73).

### Minerals and Trace Elements

#### Phosphorous

With a content of ~100 mg of phosphorous per 100 mL milk and >500 mg per 100 g cheese, dairy is an important source of phosphorous in the diet (Table 2). Although data are still scarce, it is assumed that phosphorous derived from animal foods is more bioavailable than phosphorous derived from plant-based foods, as revealed by balance studies relating phosphorous intake from dietary sources to urinary excretion (74, 75). This may be explained by the binding of phosphorous to digestible compounds in animal foods, such as proteins and phospholipids. However, phosphorous also easily forms indigestible complexes in the gastro-intestinal tract (e.g., with calcium), and its bioavailability from dairy sources may strongly depend on interaction with other meal components (74). No studies have been done to date to directly measure the bioavailability of phosphorous from dietary sources in humans.

#### Zinc

With a concentration of 0.4 mg of zinc per 100 mL milk forms an important source of zinc (Table 2). Zinc is predominantly present in the protein fraction in milk, specifically in casein micelles, but is easily released under mild acidic conditions (76). Approximately 25–30% of zinc is absorbed from milk (77, 78). Apart from whey and casein peptides, also other low molecular ligands and chelators that can bind Zn, such as amino acids (histidine, methionine) and organic acids (citric, malic and lactic acid), may promote zinc absorption (79).

#### Magnesium

Milk contains ~10 mg of magnesium per 100 mL, but can be triple that in cheese (Table 2). Absorption of magnesium from milk was found to be strongly dose-dependent, with ~75% absorption reported from a serving of milk containing 46 mg of magnesium (80). With intake of magnesium at physiological doses, absorption seems to be predominantly due to a saturable mechanism and at higher amounts mainly by simple diffusion (80). As for other divalent metals, i.e., calcium, iron and zinc, peptides from casein or whey can bind magnesium, which may promote absorption (81). Also, lactose appeared to promote absorption of magnesium from milk in rats (81, 82), but this was not confirmed in humans (83). As for calcium, unabsorbed lactose may act as a prebiotic and stimulate magnesium uptake in the large intestine, but this needs further investigation (84, 85).

#### Iodine

Iodine content of milk can vary substantially with a reported range of 3.3–53.4 μg per 100 mL, depending on the way of farming, iodine intake of dairy cows, use of iodine-containing udder cleansers, season, and processing (86, 87)_._ Iodine in milk is predominantly (>80%) present as inorganic iodide, and in line with this iodine bioavailability from milk is high (~90%) (88).

## Vegetables and Fruits

Vegetables and fruits form a widely diverse food group that contains a broad range of essential nutrients. Vegetables and fruits are generally low in fat and proteins and therefore contribute relatively little to energy intake. Ample consumption of vegetables and fruits is promoted worldwide. Such recommendations are based on studies consistently showing that higher intake of vegetables and fruits is negatively associated with all-cause mortality and mortality from cardiovascular disease and cancer (89, 90). Close to 75% of the world population consumes less than the recommended 400 g of vegetables and fruits on a daily basis (91). Low consumption of vegetables and fruits is estimated to contribute 1.8% to the total global burden of disease, primarily through cardiovascular diseases and cancer (92).

So far, studies have failed to attribute the healthful effects of vegetables and fruits to any of its isolated components. Therefore, health benefits from vegetable and fruit consumption are rather to be explained as the resultant of additive and synergistic effects of its components (63–66). They are a particular rich source of pro-vitamin A carotenoids, vitamin C, folate, vitamin K-1, potassium, calcium, magnesium, iron, and several other trace elements (14, 93)^2^.

Non-nutritive bioactive compounds are also present in multitude, comprising of phenolics, carotenoids, and glucosinolates. Although these bioactive compounds are regarded as non-essential for human survival, they may exert health effects such as reduced risk of non-communicable and degenerative diseases (71–76). Delivery of fiber, both digestible and indigestible, is another important nutritional aspect of vegetables and fruits. It has an important impact on satiety, gastrointestinal processing, metabolic parameters, and microbiota composition. It constitutes a group of heterogeneous polymers such as non-starch polysaccharides, cellulose, resistant starch, inulin, lignins, chitins, pectin, beta-glucans, and oligosaccharides. Dietary fiber may stimulate intestinal fermentation, thereby altering the production of microbial phenolic metabolites and enhancing mineral absorption (94, 95). However, dietary fiber can also negatively affect the absorption of nutrients because of gel formation, increased viscosity, or binding and entrapment (96–98). Other compounds present in vegetables and fruits may have negative consequences for human nutrition and health, such as alkaloids, oxalates, phytic acid, lectins, trypsin and protease inhibitors, tannins, and cyanogens. Anti-nutrients can be removed or inactivated by various food processing procedures, such as fermentation, germination, boiling, leaching, and extraction (99).

## Vegetables and Fruit as Sources of Nutrients

### Vitamins

#### Pro-Vitamin a Carotenoids

β-carotene, α-carotene and β-cryptoxanthin are the most common dietary carotenoids that can be converted to vitamin A (retinol) through central cleavage by β-carotene monooxygenase (bco1). B-carotene has the highest affinity to the cleavage enzyme, and, based on its chemical structure, can provide twice as much retinol as compared to the other two carotenoids. Therefore, and also because it is more abundant in the diet, β-carotene has received the most attention in vitamin A research. Liberation of β-carotene from the fruit or vegetable matrix is one of the main limiting steps in its bioavailability (100, 101). Green leafy vegetables, such as spinach and kale, are rich in β-carotene (Table 3), but only around 5–10% of the total content is bioavailable. In contrast, β-carotene from fruits show higher bioavailability despite their relatively lower β-carotene content (102, 103). This is explained by the digestibility of the particular plant compartment where the β-carotene is stored. Notably, green leafy vegetables store β-carotene in chloroplasts, which is not easily digestible for humans, whereas mangoes, for instance, store β-carotene in chromoplasts from which it is more readily available. Moreover, β-carotene in its crystallized form, as found in carrots, is not easily absorbed, in contrast to β-carotene present in lipid droplets as found in papaya (102, 103). The amount (μg) of β-carotene required to form 1 μg of retinol is referred to as conversion factor; this is estimated as 2.1–3.8 μg of β-carotene when it is provided as a supplement dissolved in oil (Table 4). Conversion factors for β-carotene from a wide variety of vegetables and fruits have been comprehensively summarized (104). In contrast to the earlier retinol equivalents (RE) which assumed that intake of 6 μg of β-carotene would yield 1 μg of retinol, current insights have shown that the bio-conversion efficiency is much lower for an average western diet. Therefore, new retinol activity equivalents (RAE) for β-carotene have been set at 12:1 (105). Conversion efficiency of α-carotene and β-cryptoxanthin have hardly been studied, although lately there is renewed interest in the latter (106, 107). Fat content of the diet is the most important enhancer of carotenoid absorption (108–110), whereas fiber present in the diet can reduce absorption efficiency (96).

**Table 3:** Bioavailability of vitamins and minerals from vegetables and fruits in humans.

**Nutrient**	**PRI/AI**	**Content (per 100 g)**		**Bioavailability (%)**	**Enhancing factors**	**Inhibiting factors**
(Pro)-vitamin A	650 μg/d	Carrot Kale Mango Orange	694 μg 335 μg 26 μg 8 μg	0–36%	Lipid droplets Dietary fat	Entrapment in cell matrix/ structures Crystallization Dietary fiber
Folate	330 μg/d	Spinach Broccoli Orange Banana	130 μg 77 μg 33 μg 9 μg	60–98%	5-methyl tetrahydrofolate vitamer	Presence of polyglutamate chain
Vitamin C	95 mg/d	Kale Broccoli Orange Kiwi	100 mg 47 mg 51 mg 79 mg	80–90%	Vitamin E	Flavonoids
Vitamin K	70 μg/d	Kale Spinach Kiwi	623 μg 394 μg 11 μg	5%	Fermentation products	Entrapment in cell matrix/ structures
Potassium	3,500 mg/d	Spinach Kale Banana Kiwi	539 mg 400 mg 374 mg 312 mg	60–85%		Food matrix of unprocessed vegetables and fruits
Calcium	950 mg/d	Kale Spinach Kiwi Orange	180 mg 105 mg 30 mg 23 mg	20–40%		Phytate Oxalate
Magnesium	300 mg/d	Spinach Kale Banana Kiwi	55 mg 34 mg 28 mg 14 mg	25–35%	Proteins Medium chain triglycerides Indigestible carbohydrates	Phytate Oxalate Cellulose Lignin Pectin
Iron	11 mg/d	Spinach Kale Broccoli Kiwi	2 mg 1 mg 0.6 mg 0.5 mg	~12%	Vitamin C Lactic fermentation	Entrapment in cell matrix and structures Phytic acid

*PRI, Population Reference Intake; AI, Adequate Intake for adult females. Source: EFSA: https://www.efsa.europa.eu/sites/default/files/assets/DRV_Summary_tables_jan_17.pdf*.

**Table 4:** Reported conversion factors and bioefficacy for β-carotene from vegetables and fruits.

**Food**	**Conversion factor^**a**^**	**Bioefficacy (%)^**b**^**
Synthetic β-carotene in oil	2.1:1–3.8:1	26–48
Fruits	12:1	8.3
Tubers	2.8:1–13.4:1	7.5–36
Cooked vegetables	10:1–28:1	1–3.5
Raw vegetables	13:1–77:1	0.01–7.7

a *Amount (μg) of ingested β-carotene required from the food source to form 1 μg of retinol*.

b *Proportion of ingested β-carotene that is absorbed and converted to retinol. Based on: Van Loo-Bouwman et al., Br J Nutr 2014 (104)*.

#### Folate

Green leafy vegetables and citrus fruits are important dietary sources of folate (Table 3). In vegetables and fruits, folate is mostly present in its polyglutamated form. Before absorption, enzymatic cleavage of this glutamate chain by folylpoly γ-glutamyl carboxypeptidase (FGCP) is necessary. It has been shown that, as compared to supplemental folic acid, which is a monoglutamate, polyglutamated folate has a bioavailability of ~70% (111, 112). Others have shown that 5-methyl-tetrahydrofolate is the best bioavailable natural form of the vitamin (113). Folate bioavailability ranges between 60 and 98% from a diet high in vegetables and fruits (91). Whereas, the food matrix, dietary fiber, and low pH may inhibit folate bioavailability, zinc enhances FGCP activity and therefore would promote folate absorption (114). Dietary folate equivalents (DFE) have been defined as 1.7 μg of dietary folate to deliver 1 μg of folate to the body circulation (115).

#### Vitamin C

Certain fruits, such as kiwi and orange, but also many vegetables are rich sources of vitamin C (Table 3). Unlike some other vitamins, vitamin C derived from vegetables and fruits largely shows similar bioavailability as compared to synthetic vitamin C at 80–90% in human studies (116–118). Nevertheless, entrapment of vitamin C in the food matrix, premature degradation or inhibition by other food components may decrease its bioavailability. Vitamin C interacts with vitamin E by reducing tocopheroxyl radicals; vice versa, vitamin E might preserve vitamin C *in vivo* (119). Although it is uncertain if flavonoids can affect vitamin C absorption *in vivo*, several *in vitro* studies showed that flavonoids inhibit the absorption of vitamin C (120–122).

#### Vitamin K

Dark green leafy vegetables and herbs such as kale, parsley, spinach, and green cabbage (Table 3) are rich in phylloquinone (vitamin K1), whereas among the fruits kiwi and avocado by exception contain reasonable amounts as well (123, 124). Menaquinones (vitamin K2) are generally not found in vegetables and fruits, but an exception to this is fermented vegetables such as sauerkraut (124). Data on the bioavailability of phylloquinone from dietary sources are scarce, but some studies show <5% bioavailability from dark green leafy vegetables, while addition of fat or oils improves bioavailability markedly (124–126). Low bioavailability can be explained by binding of phylloquinone to the membranes of plant chloroplasts (127).

### Minerals

#### Potassium

Consumption of vegetables and fruits contributes importantly to potassium intake, especially from dark green leafy vegetables and certain fruits such as banana and kiwi (Table 3). High intake of potassium has consistently been associated with reduced blood pressure and risk for hypertension (128, 129). Potassium is almost completely absorbed from dietary sources, although matrix effects may hinder potassium absorption from unprocessed vegetables and fruits to some extent. Estimates of bioavailability range between 60 and 85% from such sources (130, 131). Little is known about factors that promote or inhibit the absorption of potassium from individual dietary sources (132).

#### Calcium

Especially dark green leafy vegetables such as kale and spinach contribute to dietary calcium intake (Table 3). Studies have shown that calcium absorption from various vegetables is either inferior or comparable to calcium absorption from milk with bioavailability estimates ranging between 20 and 40% (133–135), although *Brassica* sp. vegetables showed slightly higher absorption (136). Phytate and oxalate content determine the efficiency of calcium absorption from vegetables. Phytic acid, or inositol polyphosphate, as well as oxalate, or ethanedioate, form insoluble and non-digestible complexes with divalent cations such as Fe^2+^, Zn^2+^, Ca^2+^, and Mg^2+^, which limits the bioavailability of these minerals. Oxalate is the conjugate base of oxalic acid, which is present in high amounts in certain vegetables such as spinach, cabbage, broccoli, brussels sprouts, beetroot, and rhubarb.

#### Magnesium

Magnesium can be derived in moderate amounts from fruits and vegetables (Table 3). Magnesium from dark green leafy vegetables was shown to have a bioavailability of 25–35% (137). Magnesium is assumed to be absorbed as the ion rather than as in the form of a complex (138). The absorption of magnesium is inhibited by oxalate (139). As explained for calcium above, oxalic acid can form indigestible complexes with divalent cations at physiological pH. It has been shown before that addition of oxalate-rich vegetables to the diet resulted in negative zinc and magnesium balances. Spinach, an oxalate rich vegetable, indeed showed lower magnesium bioavailability as compared to kale, a vegetable low in oxalate (137). Other known dietary based inhibitors of magnesium absorption are phytic acid, cellulose, lignin, and possibly pectin, whereas proteins, medium chain triglycerides, and indigestible carbohydrates are among the enhancers (139).

#### Iron

Green leafy vegetables are rich in iron (Table 3), but the bioavailability of iron is relatively low—around 12% (140). The low bioavailability is attributed to the indigestibility of cellular components such as chloroplasts and mitochondria where iron is stored (141). Vitamin C is well-known to aid non-heme iron bioavailability, either by enhancing iron solubility or by acting as a co-factor in the reduction of iron from the ferric to the ferrous form by duodenal cytochrome B (142, 143). Fytic acid is a strong inhibitor of iron absorption (144), whereas the inhibiting properties of oxalate are less clear. One study showed that oxalic acid did not reduce iron absorption from kale (145). A study in human volunteers showed that lactic fermentation of vegetables doubled iron absorption, which was explained by the acidic conditions that promote the presence of ferric iron, which is more stable in the gastrointestinal tract (146).

## Conclusion

Both milk as well as vegetables and fruits are nutrient-dense foods that provide a myriad of nutrients which impact human metabolism and health. Bioavailability is an important explanatory step between the food source and potential health effects of its food components. Much of the health benefits of foods may be explained by additive, antagonistic and synergistic processes at the level of uptake and absorption of nutrients. As has become clear from this review, bioavailability values from whole foods have been established in humans for some nutrients, but are still lacking or need confirmation for others. Translation of this information to individual diet scores will require detailed dietary intake information, preferably at the meal level, while taking information on bioavailability of nutrients from separate foods as well as food-to-food interactions into account. This is all the more complex, since bioavailability estimates are currently already incorporated into dietary reference intakes at the population (group) level to a certain extent. Furthermore, host-related factors, e.g., nutrient status, disease state and genetics, also play an important role in nutrient uptake and bioavailability at the individual level and are often unknown. Nevertheless, accounting for nutrient bioavailability based on food intake pattern may result in better estimates of true individual absorbable nutrient intake in relation to health outcomes. Moreover, such knowledge may help in the design of foods, meals and diets that aid in the supply of nutrients to specific target groups.

## Author Contributions

AM-B conducted the literature review and wrote the manuscript.

## Conflict of Interest

The author declares that the research was conducted in the absence of any commercial or financial relationships that could be construed as a potential conflict of interest. The handling editor declared a past co-authorship with the author AM-B.

## Footnotes

^1^https://www.globaldietarydatabase.org/our-data/data-visualizations/dietary-data-region

^2^https://wateetnederland.nl/resultaten/vitamines-en-mineralen/bronnen
